# Surgical debridement of corneal shield ulcers in pediatric patients: two case reports and a review of the literature

**DOI:** 10.1186/s13256-020-02407-8

**Published:** 2020-06-17

**Authors:** Ricardo Alexandre Stock, Silvia Letícia Teixeira Lazzari, Isadora Proner Martins, Elcio Luiz Bonamigo

**Affiliations:** 1 Ophthalmology, University of the West of Santa Catarina (Universidade do Oeste de Santa Catarina–UNOESC), Joaçaba, SC Brazil; 2 Belotto Stock Centro Oftalmológico, Rua Rio Branco, 589, Centro, CEP: 89., Joaçaba, SC 600-000 Brazil; 3 Department of Medicine, UNOESC, Joaçaba, SC Brazil

**Keywords:** Vernal keratoconjunctivitis, Shield ulcer, Pediatric ophthalmology, Surgical debridement, Case report

## Abstract

**Background:**

Ocular allergy is a common disease, especially in the pediatric population, with unpleasant and long-term consequences, including corneal complications and decreased visual acuity. This study reports two cases of corneal shield ulcer due to vernal keratoconjunctivitis, with good results of surgical debridement performed after failure of long-term clinical treatment. Furthermore, this study highlights that this therapeutic approach, although less common, is efficient in treating refractory cases that cause suffering in pediatric patients.

**Case presentation:**

The first patient was a 7-year-old Caucasian boy with chronic ocular allergy, especially photophobia, who had been treated with eye drops, antihistamine, and corticosteroids for 60 days without success. Biomicroscopy of the right eye showed the presence of gelatinous limbus, giant papillae in the tarsal conjunctiva, and a shield ulcer measuring 6.0 mm vertically and 2.7 mm horizontally. Surgical debridement was performed, and the ulcer did not recur. The second patient was a 4-year-old Caucasian boy with chronic ocular allergy, especially itching and photophobia, who had been treated with eye drops for 1 year without success. Biomicroscopy of the left eye showed a shield ulcer, with a dense central corneal plaque, measuring 8 mm vertically and 3.5 mm horizontally. Surgical debridement of the ulcer was performed immediately because of the chronicity of the case and severity of the lesion, and the treatment was effective.

**Conclusions:**

The treatment of shield ulcers caused by vernal keratoconjunctivitis in the two reported cases was curative and definitive by surgical debridement in the 7-month follow-up period. Therefore, the early debridement of shield ulcers refractory to drug treatment can considerably reduce the time of disease evolution and the probability of ocular complications caused by clinical treatment or disease chronicity. However, this approach is rarely described in the literature and needs to be included in the ophthalmologist’s therapeutic arsenal.

## Background

Vernal keratoconjunctivitis is a chronic allergic disease that affects the ocular surface and is associated with a history of atopy. This disease predominantly affects the pediatric male population age 5 to 15 years [1] and usually disappears after puberty. The manifestation is usually bilateral and occurs seasonally, especially in the spring [2]. Ophthalmologic examination indicates papillary hypertrophy, Horner-Trantas dots, and, rarely, corneal shield ulcer; the latter manifestation is one of the most severe complications of this disease and can progress to loss of vision [3, 4].

Two hypotheses can explain the development of shield ulcers. The first is the mechanical friction generated by the giant papillae, causing a micro-corneal trauma that later evolves into a shield ulcer. The second is an allergic response produced by the toxic action of inflammatory mediators released by eosinophils [5].

Several types of shield ulcer treatments have been proposed and studied, including topical immunosuppressive agents (corticosteroids, cyclosporine, and tacrolimus), non-steroidal anti-inflammatory drugs, homeopathic medications, surgical debridement of the corneal plaque to remove cytotoxic cells, and amniotic membrane transplantation [3, 6]. New drugs are being studied and can potentially be used in treatment [7]. However, the predominance of clinical approaches warrants further study of early surgical debridement, which may be an effective treatment option because of its ability to rapidly interrupt the course of the disease [8, 9], as demonstrated in these two cases. In addition, the timing of the surgical procedure is crucial because it affects pediatric patients, for example, high levels of amblyopia and strabismus are associated with delayed treatment.

## Case presentation

### Case 1

A 7-year-old healthy Caucasian boy with no family history of shield ulcers presented with chronic ocular allergy in both eyes. He complained of eye burning, foreign body sensation, itching, and photophobia. He was treated with olopatadine hydrochloride (1.11 mg/mL) and dexamethasone (1 mg/mL) for 60 days without success.

**Fig. 1:**
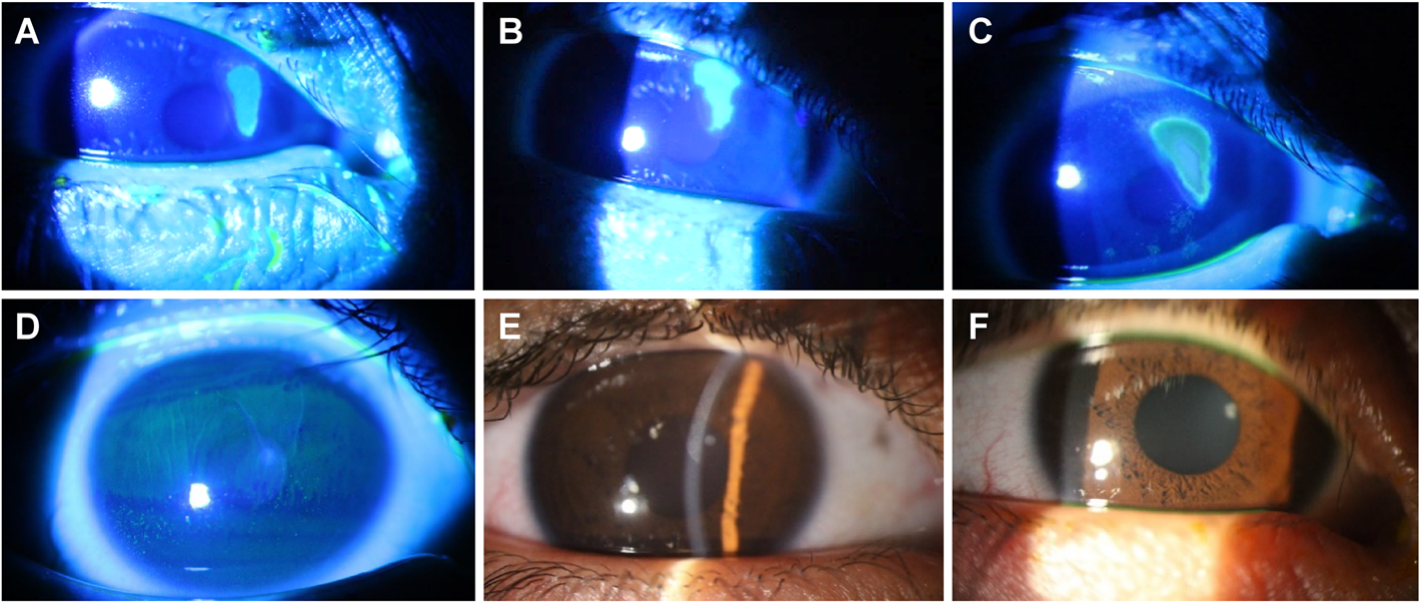
Evolution of the shield ulcer in the right eye. Right cornea with a shield ulcer measuring 6.0 mm vertically and 2.7 mm horizontally (**a**). The ulcer persisted after drug treatment (**b**). Increase in the epithelial defect after maintenance of drug treatment for 90 days, and the defect returned to the size obtained in the first evaluation (**c**). Clinical picture 7 days after surgical debridement, with complete re-epithelialization of the cornea (**d**). Slight opacity in the anterior stroma in the area of the previous ulcer (**e**). Slight opacity in the anterior stroma 10 months after surgical removal of the plaque (**f**)

A physical examination showed a corrected visual acuity (VA) of 20/40 in the right eye (RE) and 20/125 in the left eye (LE). Biomicroscopy of the RE revealed the presence of gelatinous limbus, giant papillae in the tarsal conjunctiva, and a grade 2 shield ulcer with a dimension of 6.0 mm vertically and 2.7 mm horizontally (Fig. 1a). The LE presented gelatinous limbus, giant papillae in the tarsal conjunctiva, and diffuse keratitis. Fundoscopy was unremarkable in both eyes.

Treatment with prednisolone acetate 1% twice daily and the antihistamine epinastine hydrochloride 0.05 mg/ml eye drops twice daily was started in both eyes to fight allergy, with the recommendation to use cold compresses and avoid scratching the eye. After 7 days of treatment rigorously accomplished, our patient presented improvement in the clinical picture. The corrected VA was 20/20 in both eyes. A biomicroscopy of the RE indicated the absence of gelatinous limbus and the presence of giant papillae in the tarsal conjunctiva but no improvement in the shield ulcer. Superficial keratitis was observed in the LE. The intraocular pressure (IOP) was 14 mmHg in both eyes, and drug treatment was maintained.

In the following consultation after 3 weeks, the corrected VA was 20/20 in both eyes, with a slight reduction in the shield ulcer size (Fig. 1b). The LE was unremarkable, and the IOP was 14 mmHg in both eyes. In view of the poor improvement of the ulcer, surgical removal of the plaque at the base of the shield ulcer was indicated in a surgical environment but was not accepted by the parents. In the following consultation after 90 days, the epithelial defect and plaque were larger (Fig. 1c). The corrected VA was 20/40 in the RE and 20/20 in the LE, with an IOP of 14 mmHg in both eyes. Two weeks later, surgical debridement was successfully performed in a sterile surgical environment under intravenously administered sedation and anesthetic support. After the procedure, an occlusive dressing with ciprofloxacin hydrochloride ointment (3.5 mg/g) and dexamethasone (1.0 mg/g) was applied for a week.

One week after the procedure, our patient returned without complaints. The RE presented a completely re-epithelialized cornea with a diffuse anterior stromal haze, and the IOP was 14 mmHg in both eyes (Fig. 1d). The corrected VA was 20/20 in both eyes. Treatment with prednisolone acetate 1% every 2 days in both eyes and epinastine hydrochloride 0.05% twice daily in both eyes was prescribed, and general recommendations were given.

Forty-five days after the procedure, the child presented no ocular complaints or ocular itching. The corrected VA was 20/20 in both eyes. A biomicroscopy of the RE revealed diffuse anterior stromal haze in the area of the previous ulcer (Fig. 1e), with no detectable changes in the limbus and tarsal conjunctiva. The LE was unremarkable. Treatment was initiated with tacrolimus 0.02% twice daily in both eyes and epinastine hydrochloride 0.05% twice daily in both eyes, and general recommendations were provided.

He returned after 8 months and was prescribed 0.02% tacrolimus and epinastine hydrochloride twice daily in both eyes. There were no eye complaints. The corrected VA was 20/20 in both eyes (Table 1). Biomicroscopy of the RE revealed diffuse anterior stromal haze (Fig. 1f).

**Table 1:** Timeline of case 1

Timeline	Description of the presentation and follow-up	Duration of topical medication and ulcer resolution
Day 1	Patient with grade 2 shield ulcer in the right eye. Treatment with prednisolone acetate 1% twice daily and epinastine hydrochloride 0.05 mg/ml eye drops twice daily	Start of assistance with topical medication
Day 7	No improvement in the shield ulcer	Maintenance of topical medication
Day 28	Slight reduction in the shield ulcer. Surgical removal of the plaque was indicated. Lost contact with the patient	After 28 days, the assistance was interrupted for 104 days
Day 118	Return of the patient. Treatment with prednisolone acetate 1% twice daily and epinastine hydrochloride 0.05 mg/ml eye drops twice daily	Restart of topical medication for 14 days prior to surgery
Day 132	Surgical debridement was performed. Occlusive dressing with ciprofloxacin hydrochloride ointment (3.5 mg/g) and dexamethasone (1.0 mg/g) was applied for a week	New topical medication for 7 days after surgery and ulcer resolution in this period
Day 139	Completely re-epithelialized cornea with a diffuse anterior stromal haze. Treatment with prednisolone acetate 1% every 2 days in both eyes and epinastine hydrochloride 0.05% twice daily	Topical treatment changed and maintained for 45 days
Day 184	Diffuse anterior stromal haze in the area of the previous ulcer. Treatment with tacrolimus 0.02% twice daily in both eyes and epinastine hydrochloride 0.05% twice daily in both eyes	Topical medication maintained for another 2 months
Month 8	Diffuse anterior stromal haze, with no signs of ulcer. Clinical treatment maintained	Topical treatment maintained

### Case 2

**Fig. 2:**
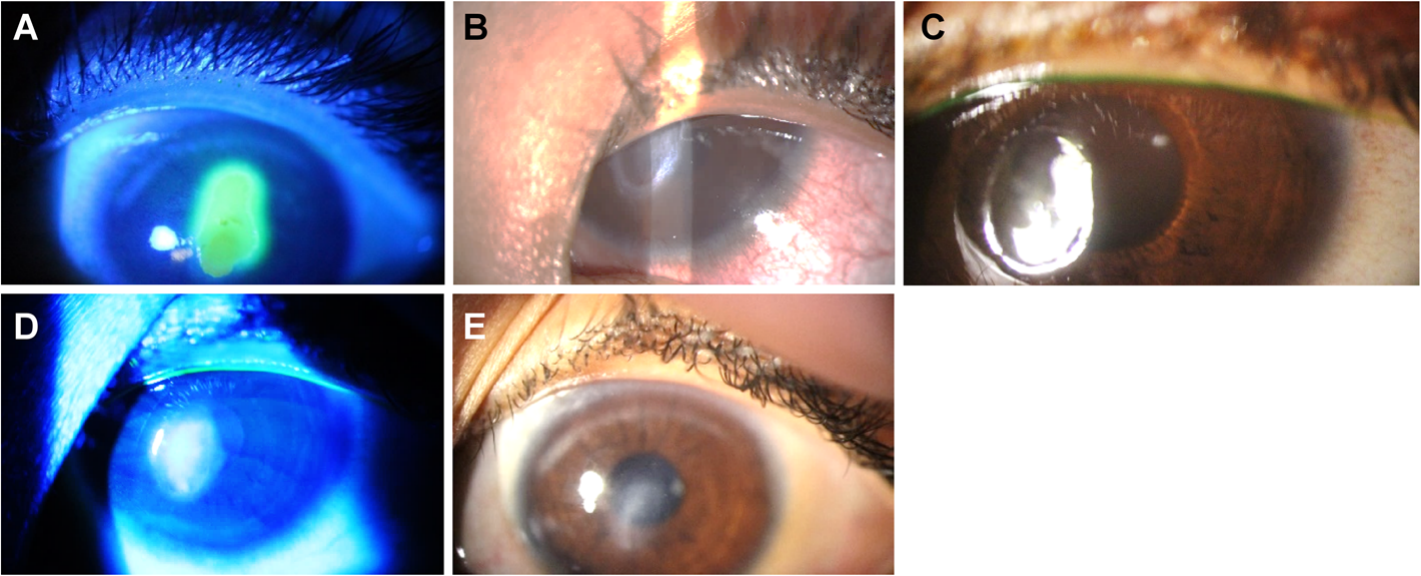
Evolution of shield ulcer in the left eye. Left cornea with a shield ulcer measuring 8.0 mm vertically and 3.5 mm horizontally, with a dense plaque at the base of the lesion (**a**). Large epithelial/anterior stromal defect at the debridement site on the first postoperative day (**b**). Complete re-epithelialization of the cornea 2 weeks after surgical debridement with anterior stromal thinning (**c**). Moderate diffuse central anterior stromal haze 45 days after surgical debridement without epithelial defect (**d**). Diffuse central anterior stromal haze 8 months after surgical debridement without epithelial defect or relapse (**e**)

A 4-year-old healthy Caucasian boy, who was the son of healthy parents, presented chronic allergy in both eyes for 1 year, especially itching and photophobia, and underwent drug treatment without success. RE biomicroscopy showed giant papillae in the upper tarsal conjunctiva, papillae in the lower tarsal conjunctiva, and normal cornea. The LE presented a shield ulcer (classified as grade 3) with dense plaque in the central area of the cornea, measuring 8.0 mm vertically and 3.5 mm horizontally, without signs of infection (Fig. 2a). It was not possible to determine the VA and IOP because of patient non-compliance. Topical treatment was initiated with prednisolone acetate 1% every 4 hours in both eyes and 0.05% epinastine hydrochloride twice daily in both eyes. Surgical removal of the plaque was performed immediately because of the chronicity of the condition and severity of the lesion.

Our patient presented no pain on the first postoperative day. RE biomicroscopy was unremarkable, and the LE showed a large epithelial/anterior stromal defect at the debridement site, with no other changes (Fig. 2b). Topical treatment was maintained with prednisolone acetate 1% every 4 hours and epinastine hydrochloride 0.05% every 12 hours in the LE. Our patient was treated with occlusive dressings containing ciprofloxacin hydrochloride ointment (3.5 mg/g) and dexamethasone (1.0 mg/g), for a week.

In the follow-up after 5 days, the parents reported applying an occlusive dressing to our patient’s LE for 24 hours daily. The child presented no ocular pain or itching. Biomicroscopy of the RE was unremarkable. A central epithelial defect (1.5 mm vertically and 0.3 mm horizontally) was observed in the LE with no signs of infection. An occlusive dressing with ciprofloxacin hydrochloride ointment (3.5 mg/g) and dexamethasone (1.0 mg/g) was applied to the LE daily for one more week, until total re-epithelization.

Two weeks after the procedure, the child presented no complaints, and occlusive dressings were applied to the LE for 24 hours daily. Biomicroscopy of the LE showed a 20% anterior stromal thinning in the absence of fluorescein staining, dye pooling, complete re-epithelialization of the lesion, and no signs of infection (Fig. 2c). Forty-five days following the procedure, the child presented no complaints, and treatment compliance was excellent (Fig. 2d). Topical corticosteroid treatment was suspended, and treatment was initiated with tacrolimus 0.02% twice daily and epinastine hydrochloride 0.05% twice daily in both eyes. Six months after discharge, he was treated with tacrolimus 0.02% twice daily and epinastine hydrochloride 0.05% twice daily in both eyes. The uncorrected VA was 20/25 in the RE and 20/60 in the LE (Table 2). Biomicroscopy of the LE showed moderate diffuse anterior stromal haze without an epithelial defect (Fig. 2e).

**Table 2:** Timeline of case 2

Timeline	Description of the presentation and follow-up
Day 1	Chronic allergy in both eyes for 1 year and underwent drug treatment without success. Shield ulcer classified as grade 3. Surgical removal of the plaque was performed immediately
Day 2	Large epithelial/anterior stromal defect at the debridement site. Occlusive dressing with ciprofloxacin hydrochloride ointment (3.5 mg/g) and dexamethasone (1.0 mg/g) was applied for a week
Day 7	Central epithelial defect with no signs of infection. Occlusive dressing with ciprofloxacin hydrochloride ointment (3.5 mg/g) and dexamethasone (1.0 mg/g) was applied to the LE daily for one more week. Treatment with prednisolone acetate 1% twice daily and epinastine hydrochloride 0.05 mg/ml eye drops twice daily, after a week
Day 21	Anterior stromal thinning in the absence of fluorescein staining, dye pooling, complete re-epithelialization of the lesion, and no signs of infection. The clinical treatment was maintained
Day 45	Complete re-epithelialization of the lesion. Topical corticosteroid treatment was suspended, and treatment was initiated with tacrolimus 0.02% and epinastine hydrochloride 0.05%
Month 6	Moderate diffuse anterior stromal haze without an epithelial defect. In routinely use of tacrolimus 0.02% twice daily in both eyes and epinastine hydrochloride 0.05% twice daily in both eyes

## Discussion and conclusions

Vernal keratoconjunctivitis is a subtype of chronic allergic conjunctivitis that affects the eyes, usually bilaterally, as observed in these two cases, and the prevalence is higher in the spring [5] in countries with dry and hot climates [3]. Brazil, with a tropical climate, is a favorable location for disease development. The most affected population is male children of age 5 to 15 years [1], with a proportion of three boys to one girl until puberty. After this period, men and women are equally affected [10]. Our patients were aged 4 and 7 years.

This disease has three forms of presentation: (1) the eyelid form, which is more common in Europe and the USA and affects the papillae in the upper palpebral conjunctiva, which may fuse and form giant papillae usually larger than 1 mm; (2) the bulbar/limbal form, which is more frequent in Asia and Africa and is characterized by hypertrophy of the limbal papillae, with a tendency to fuse and present a gelatinous appearance; and (3) the mixed form, which is a combination of the palpebral and limbal form and is more common in tropical countries [6]. Our cases belong to type 3 since they have a palpebral, limbal, and corneal component.

The symptoms are usually more severe and acute than those associated with seasonal conjunctivitis, as observed in the two reported cases. Ophthalmologic examination indicates hyperemia, chemosis, papillary hypertrophy, the presence of giant papillae in some cases, and Horner-Trantas dots formed by degenerating eosinophils, potentially leading to shield ulcer due to trauma or toxicity [3].

The indication for treatment of shield ulcers differs according to the degree of severity, which varies from 1 to 3 [5]. Grade 1 ulcers have a clear base and margins, no macroscopic inflammatory material, a good response to drug treatment, and rapid re-epithelialization. Grade 2 ulcers take longer to re-epithelialize because of the presence of inflammatory material at the margins and base, and the complication rates are consequently higher. In grade 3 ulcers, proteins are deposited in the lacrimal film and Bowman’s layer. Therefore, surgical treatment is indicated starting at grade 2 lesions, especially plaque removal, because short-term re-epithelialization rates are higher and the number of complications is lower than that associated with drug treatment [5, 6].

Treatment depends on several factors, including patients’ or parents’ choice, doctor’s surgical ability, the accessibility of hospital, the cost of the procedure, and ulcer progression. Drug treatment primarily involves the use of immunosuppressants, including corticosteroids and tacrolimus, non-steroidal anti-inflammatory drugs, and homeopathic medication. Treatment in the form of topical eye drops can be used in lower-grade ulcers [11] and was instituted in these two cases. However, both of our cases were treated with topical eye drops but it was not enough so surgical removal was necessary. In addition to plaque removal, amniotic membrane transplantation can be associated with drug treatment, but the technical difficulties are greater [5].

In both patients the anesthetic procedure was performed using sedation associated with topical anesthesia. With the help of a blunt spatula, the cleavage plane of the protein membrane was identified. The membrane is pulled gently with tooth tweezers, avulsed with smooth movements and removed in a single piece. Although we have presented only two cases, few studies to date have discussed the advantages of excision of shield ulcers to interrupt the course of the disease. A database search in PubMed, ScienceDirect, Scopus, Google Scholar, SciELO, and LILACS yielded the following articles on the subject (in chronological order): Solomon *et al.* [12], Ozbek *et al*. [8], Fukuda *et al*. [13], Caputo *et al*. [14], Reddy *et al*. [5], Mushtaq *et al*. [9], Cameron [15], and Das [16] (Table 3).

**Table 3:** Literature review on surgical excision of shield ulcers

Author(s)	Year	Number of patients	Age in years	Medicine used after surgery	Follow-up	Results
Cameron [15]	1995	23	Mean 12.7	Topical cell stabilizers	Variable	Effective in 20 of 23 patients
Solomon *et al*. [12]	2004	3	4, 7.5, 9	Topical steroid	8 to 15 months	Effective in all patients
Ozbek *et al.* [8]	2006	1	12	Cyclosporine 0.05%	10 months	Effective
Fukuda *et al.* [13]	2010	1	27	Fluorometholone and sodium cromoglicate eye drops	2 months	Effective
Caputo *et al.* [14]	2012	4	Children (age not available)	Cyclosporine and topical lubricating eye drops	12 months	Effective in all patients
Reddy *et al.* [5]	2013	21	Mean 12	Sodium cromoglycate 2% or 4%, prednisolone acetate 1% or fluorometholone 0.25% and lubricating eye drops	18 months	Effective in 20 of 21 patients
Mushtaq *et al.* [9]	2016	1	25	Topical steroid, mast cell stabilizers, and lubricating eye drops	2 months	Effective
Das [16]	2017	1	11	Olopatadine and lubricating eye drops	3 months	Effective

The effectiveness of plaque removal according to the identified articles is high (Table 3). If the most effective treatment is not instituted, as occurred in case 2, in which a previous drug treatment was used for an extended period, shield ulcers may progress to grade 3 [17].

Surgical treatment involves scraping the base and edges of the ulcer and removing the inflammatory plaque [4, 12]. Intraoperative optical coherence tomography-guided has been described as a method for monitoring the dissection depth of the shield ulcer with plaque [18]. In the case series presented by Cameron [15], 20 (87%) of the 23 ulcers with plaque formation exhibited rapid re-epithelialization after plaque removal, justifying the creation of an algorithm to guide treatment because only 25% of grade 2 ulcers exhibited satisfactory re-epithelialization with drug treatment alone. The results obtained by Ozbek *et al.* [8], Fukuda *et al*. [13], Mushtaq *et al*. [9], and Das [16] were excellent. Caputo *et al*. [14] found that, among 700 children under treatment, four developed shield ulcers, and they all experienced good disease resolution after plaque removal. Excimer laser phototherapeutic keratectomy was employed as an auxiliary treatment in three eyes with shield-shaped corneal ulcers and plaques caused by vernal keratoconjunctivitis [19]. Shield ulcer regression with drug treatment is rare in cases in which white or yellowish deposits develop, emphasizing the importance of surgical treatment [2, 8].

In both cases, surgical treatment was curative and definitive in the 7-month follow-up period. The risk of side effects of medications and clinical complications, including bacterial infection, emphasizes the need for surgical treatment of grade 2 and grade 3 ulcers to interrupt the course of the disease [2], as occurred in the two evaluated patients. The delay in surgical intervention may result in other complications, such as amblyopia and strabismus in pediatric patients [12]. Furthermore, as the timing of the surgical procedure is crucial, due to the high levels of amblyopia and strabismus associated with delayed treatment, we also suggest that the surgical approach should be considered the first choice in cases of types 2 and 3.

The results of these two cases provided evidence of the efficacy of surgical treatment of grade 2 and 3 shield ulcers refractory to drug treatment. In selected cases, that is, in grade 2 and 3 ulcers, surgical treatment interrupts the course of the disease and is much more effective than drug treatment, demonstrating the need to include this strategy in the therapeutic arsenal because of its immediate benefit to the patient.

The number of reported cases is small because the disease is rare. Therefore, new studies with more cases are necessary to prove the effectiveness of the described method.

## Data Availability

The author’s original image files are available within the article.

## Abbreviations

RE: Right eye
IOP: Intraocular pressure
LE: Left eye
VA: Visual acuity
